# Recent Advances in Structured Catalytic Materials Development for Conversion of Liquid Hydrocarbons into Synthesis Gas for Fuel Cell Power Generators

**DOI:** 10.3390/ma16020599

**Published:** 2023-01-08

**Authors:** Vladislav Shilov, Dmitriy Potemkin, Vladimir Rogozhnikov, Pavel Snytnikov

**Affiliations:** 1 Boreskov Institute of Catalysis SB RAS, Pr. Lavrentieva 5, 630090 Novosibirsk, Russia; 2 Faculty of Natural Science, Novosibirsk State University, Pirogova St., 2, 630090 Novosibirsk, Russia

**Keywords:** fuel cell, reforming, fuel processing, liquid fuel, diesel fuel, hydrogen, catalyst, syngas

## Abstract

The paper considers the current state of research and development of composite structured catalysts for the oxidative conversion of liquid hydrocarbons into synthesis gas for fuel cell feeding and gives more detailed information about recent advances in the Boreskov Institute of Catalysis. The main factors affecting the progress of the target reaction and side reactions leading to catalyst deactivation are discussed. The properties of the Rh/Ce_0.75_Zr_0.25_O_2_/Al_2_O_3_/FeCrAl composite multifunctional catalyst for the conversion of diesel fuel into synthesis gas are described. The results of the catalyst testing and mathematical modeling of the process of diesel fuel steam–air conversion into synthesis gas are reported.

## 1. Introduction

It was expected in most of the research work carried out over the past 20–25 years that by the time of mass use of fuel cells, the infrastructure for their supply with fuel (hydrogen) would already be created, including efficient logistics, a fully developed hydrogen refueling network and sufficient hydrogen long-time storing capacities. However, these forecasts appeared overly optimistic. Even regarding widely used natural gas, the existing and actively developing infrastructure for its transportation and consumption turns to be insufficient to cover completely the current level of mass demand.

That is why all the world’s research centers involved in R&D of fuel-cell-based power units show interest in using fuel of common types, such as natural gas, liquefied petroleum gas, gasoline, aviation kerosene, diesel fuel, methanol, ethanol, etc. Besides, compared to other currently available hydrogen storage technologies, hydrocarbon fuels demonstrate the highest hydrogen content per unit volume [1].

**Figure 1:**
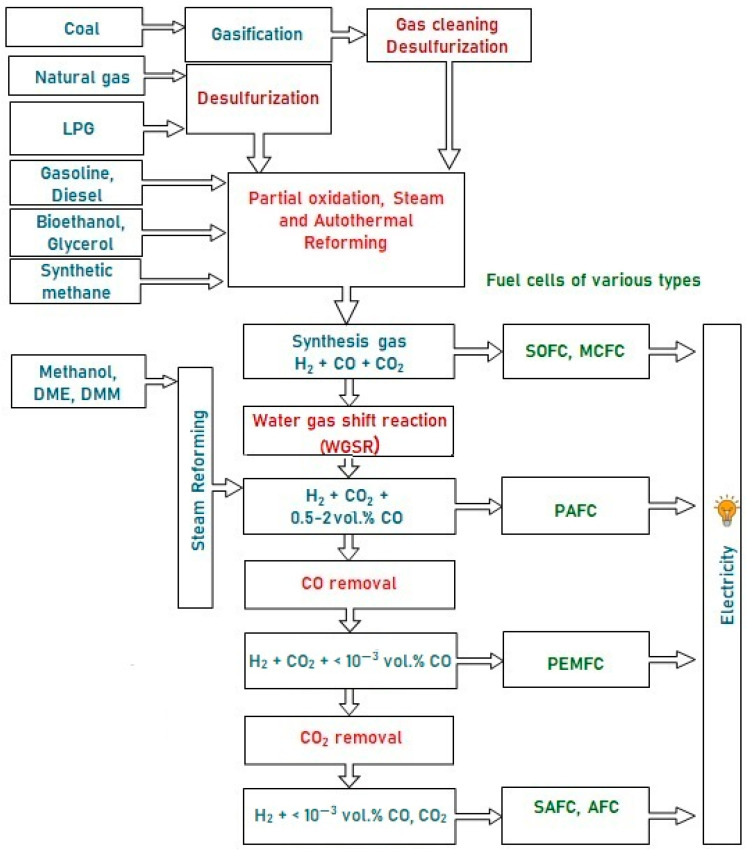
A typical scheme of hydrogen production from various fuels for feeding fuel cells of different types.

These fuels can hardly be oxidized directly in the anode space of a fuel cell (FC), since they are inert at low temperatures, and can initiate electrode coking and FC failure at high temperatures. Therefore, the fuel is first converted into a hydrogen-rich gas, which is then oxidized in a fuel cell. Depending on the FC type, hydrogen for FC fueling can contain some amounts of CO and CO_2_. Figure 1 presents a typical scheme of catalytic processes for the production and cleanup of hydrogen-rich gas mixtures from various fuels for fuel cell feeding. Note that sulfur-containing compounds are poisonous for FC of all types; therefore, any fuel must be desulfurized either at the stage of production or prior to using it for hydrogen production. The processes for liquid and gaseous fuels desulfurization are well-developed and widely used in industry, but remain beyond consideration in this review.

Synthesis gas is produced by the processes of partial oxidation (PO) (1), steam reforming (SR) (2) and autothermal reforming (ATR) (3). The most efficient process is the SR of hydrocarbons or alcohols, which, depending on the fuel type, is performed at temperatures of 300–900 °C, with excess water (molar ratio H_2_O/C = 1.1–5), and provides the highest hydrogen yield. However, since SR is an endothermic process and therefore requires a significant heat supply, a steam-generating system and water conditioning, it is usually realized in combination with stationary power plants based on high-power FCs.

(1) $$C_{n}H_{m}+\frac{n}{2}O_{2}\;\to \;\mathrm{nCO}+\frac{m}{2}H_{2}\;\;\;\;\;\;\Delta H<0$$

(2) $$C_{n}H_{m}+nH_{2}O\;\to \;\mathrm{nCO}+[\frac{m}{2}+n]H_{2}\;\;\;\;\;\;\Delta H>0$$

(3) $$C_{n}Hm_{\;}+\frac{n}{4}O_{2}\;+\frac{n}{2}H_{2}O\;\to \;\mathrm{nCO}+[\frac{m}{2}+\frac{n}{2}]H_{2}\;\;\;\;\;\;\Delta H\leq 0$$

For mobile decentralized power systems based on low-power fuel cells, the hydrogen-rich gas generating unit must be compact, highly productive and water-independent, and operate at moderate temperatures. In this regard, the PO and ATR processes are of particular interest. 

Besides the R&D studies on the conversion of natural gas and propane–butane mixtures, considerable interest is focused on the use of the main logistics fuels (gasoline, aviation kerosene and diesel fuel), based on the concept of hybrid systems, when fuel cells serve as an auxiliary power unit supplementary to the main power system—a truck diesel engine, aircraft turbines, etc. This approach promotes the efficiency of fuel consumption in modes when the main engine is either turned off or idling during stops, loading/unloading operations in cargo terminals, airport parking, etc. [2,3,4,5,6].

Oxidative conversion (SR, ATR and PO) of liquid hydrocarbons into synthesis gas e is based on the process of steam reforming of naphtha, which has been used since the 1960s in the petrochemical and ammonia industries for the production of synthesis gas and hydrogen [7]. The low sulfur content (≤0.001 wt.%) in diesel fuel of modern grades greatly promoted the development of fuel processors [3,8,9,10]. Indeed, over the past decade, the number of R&D works aimed at finding and studying catalysts for the conversion of kerosene [3,4,11,12,13], diesel [14,15,16,17], renewable natural raw materials—biodiesel [18,19] and glycerin (a by-product of biomass processing) [20]—as well as fuel processor designing [5,6,13], has increased significantly [2,4,5,6,11,13,19,20,21,22,23,24,25,26,27,28,29,30,31].

In contrast to monofuels (methanol, dimethyl ether, ethanol, etc.), which are also considered as promising raw materials for the production of hydrogen (hydrogen-rich gas) [32], the middle distillates—a product of oil refining (kerosene and diesel fuel)—are multi-component mixtures, mainly containing saturated and aromatic hydrocarbons, that seriously complicate both the study of the reforming processes and comparative analysis of the results reported by various research teams. It should be taken into account that the fuel composition can vary greatly depending on the initial oil feedstock and oil refining technologies, as well as on the individual characteristics of fuel production (including seasonal ones) [33,34]. To exclude this ambiguity, laboratory studies are mainly carried out with the use of model mixtures simulating diesel fuel or aviation kerosene chemical characteristics and the important physical properties affecting the heat and mass transfer processes. This approach simplifies the experiments, promotes the study of how individual fuel components affect the conversion process and provides comparative analysis of the catalysts’ activity.

Dodecane [35,36,37,38,39,40,41], tetradecane [42,43,44,45] or hexadecane [46,47,48,49,50,51,52,53] were often used as model substances in the studies of SR, PO and ATR processes. The effect of chemical additives of other classes (for example, toluene, alkylbenzenes, decalin, tetralin, naphthalene, 1-methylnaphthalene, simulating cycloalkanes, aromatics and polyaromatics) was studied as well [36,37,43,44,46,54]. The tests showed that, compared to paraffins and cycloparaffins, aromatic compounds are much harder to convert to synthesis gas and facilitate carbon formation [52,55,56,57,58,59]. In addition, in the presence of aromatic compounds, the rate of heterogeneous reactions of paraffin conversion decreases; that facilitates non-catalytic homogeneous processes, releasing ethylene, which also accelerates the catalyst coking [60].

In a number of works and experiments were carried out using synthetic commercial fuels and mixtures—for example, Norpar13 (ExxonMobil, Spring, TX, USA) [61], consisting of saturated hydrocarbons with an average number of carbon atoms equal to 13, NExBTL biodiesel (Neste Oil, Espoo, Finland) [14,62], GTL diesel (Shell MDS, Bintulu, Malaysia) [4], EcoPar diesel (EcoPar, Gothenburg, Sweden) [4] and GTL kerosene (Shell MDS, Bintulu, Malaysia) [4]. All these fuels are characterized by extremely low content of aromatic and sulfurous compounds—less than 0.1 and 0.0001 wt.%, respectively. So, it is no wonder that the best reported results were achieved with the use of exactly these fuels: experiments in the presence of Rh-based catalysts under appropriate reaction conditions demonstrated a 100% fuel conversion, high yield of the main reaction products (H_2_ and CO), negligible content of C_2+_ byproducts and stable operation of the catalyst for several thousand hours. The results of experiments with the fuels produced at oil refineries by traditional methods—MK1 diesel (Shell, Stockholm, Sweden) [14,17,62,63,64], SD10 (Preem, Stockholm, Sweden) [27,65], Ultimate diesel (ARAL, Hamburg, Germany) [4,17,63,64,66], automotive diesel [27] or Jet-A aviation kerosene (after additional desulphurization) [4,27]—were less impressive. The sulfur content in these fuels was a few ppm, the content of aromatics—5 (MK1), 13–18 (SD10, Ultimate diesel, Jet-A) and 24 wt.% (automotive diesel). The presence of aromatics significantly complicated the process of fuel conversion and worsened its characteristics: the fuel converted incompletely, oily residue was observed on the surface of the aqueous condensate (as the reaction is always carried out in a significant excess of steam against stoichiometry), the content of C_2+_ byproducts in the gas phase was high and carbon deposits were formed both on the catalyst and on the structural elements of the fuel reformer. To prevent undesirable reaction routes, engineering solutions were proposed, including the use of various types of nozzles, ultrasonic sprayers, specially designed evaporation and mixing chambers for homogenization of the reaction mixture before supplying to the catalyst [4,63,67,68].

Significant attention was paid to the development of structured catalysts for oxidative conversion (SR, ATR, and PO) of liquid hydrocarbons. The approaches were mainly based on the experience of creating catalytic afterburners for automobile exhausts. Cordierite blocks were used for supporting an active catalytic layer. A large number of various catalytic systems have been studied, mainly based on Rh, Ru, Pd, Pt, Ni and Co, and their bimetallic compositions in combination with various supports comprised of individual or mixed oxides of Zr, Ce, Gd, La, Y, Pr and Al, doped with alkali and alkali-earth metals; the systems supported on perovskite and pyrochlore were studied [14,15,16,17,39,42,43,45,49,52,53,54,65,69,70,71,72,73,74,75,76,77,78,79,80,81,82,83].

Although Ni-based catalysts are widely used in industrial reactors for SR of natural gas into synthesis gas, their use for the conversion of diesel fuel and aviation kerosene seems to be rather problematic. The authors of review [83] compared and analyzed the results of studies of a large number of nickel catalysts in the reactions of partial oxidation and carbon dioxide conversion of methane, and concluded that although the use of a Ce_1-x_Zr_x_O_2_ mixed oxide, possessing high oxygen mobility, as a support, the introduction of perovskite BaTiO_3_, characterized by a large number of oxygen vacancies, into the catalyst composition and the catalyst doping with K, Ca, Y, La and Pr oxides all contributed to an increase in catalyst activity compared to Ni/Al_2_O_3_, these factors appeared unable to impede completely the processes of carbon formation, and the catalysts suffered rapid coking. Nevertheless, many research teams around the world persistently undertake attempts to create active and stable catalysts for the pre-reforming and steam reforming of diesel and kerosene fuels [16,54,73,84,85,86,87,88].

Comparative studies of noble-metal-containing catalysts showed that the Pt-, Ru- and Pd-based systems stood behind the Rh-based ones in catalytic activity, stability and coking resistance [39,40,49,52,53], and therefore these metals were considered mainly as doping additives. It should be noted that, even in laboratory experiments, the catalyst depositing (coating) on substrates with high thermal conductivity is a necessary trick for preventing undesirable hot spot formation in the catalyst, which can accelerate coking processes. Laboratory studies showed that the Rh- and Rh-Pt-based ceramic block catalysts exceed in activity, selectivity and stability other catalytic systems at diesel ATR [55,56,57]. Quite naturally, cordierite-supported RhPt/Al_2_O_3_-CeO_2_ commercial catalysts (Umicore AG&Co. KG, Hanau, Germany) were used in pilot tests of more than a dozen fuel reformer modifications performed by the research team from Forschungszentrum Jülich (Jülich, Germany) [4,5,6]. To reduce temperature heterogeneity at diesel ATR, induced by the high exothermic effect of oxidation reactions, proceeding predominantly in the frontal zone of the catalytic block, and the high endothermic effect of the reactions of steam and carbon dioxide reforming, which take place in its tail section, it seems reasonable to use, instead of cordierite ceramics, a metal support composed of FeCrAl wire mesh, which has a high thermal conductivity [89]. Since the coefficient of thermal expansion (CTE) of high-temperature metal alloys twice exceeds that of oxide coatings, the latter often suffer cracking and destruction during heating. This problem was addressed in recent works on the development of a catalyst for diesel fuel ATR, carried out at the Boreskov Institute of Catalysis, SB RAS [18,48,49,50,51,90,91,92,93,94,95,96,97,98,99]. An elegant approach has been proposed based on supporting a needle-shaped coating instead of a continuous catalytic layer [90]. In such a “flexible” coating, individual elements of the oxide layer can move relative to each other during heating/cooling and respective expansion/compression of the metal substrate, thus preventing degradation of the catalytic layer.

## 2. Active Component of Catalysts for the Conversion of Diesel Fuel into Synthesis Gas

At present, the main amount of commercial diesel fuel (DF) produced at refineries by the process of diesel fraction hydrotreating meets the Euro 5 standard in terms of fractional composition and the content of polycyclic aromatics and sulfur. Table 1 presents a typical average composition of diesel fuel.

As noted above, a key factor for providing efficient catalytic conversion of diesel fuel into synthesis gas is to ensure the stable operation and coking resistance of the catalyst. The most active and stable catalysts for diesel fuel conversion are Rh- and other precious metal systems supported on oxide carriers containing mobile lattice oxygen, mainly zirconium and cerium oxides. The mobile lattice oxygen participates in the oxidation of incipient carbon deposits and thus significantly improves the catalyst stability. The most active carrier in this regard is cerium oxide, but at temperatures above 600 °C it is not strongly sintered and therefore cannot be used in its pure form. So, mixed oxides of composition Ce_x_Zr_1-x_O_2-δ_ were chosen as the support, as they possess both high mobility of lattice oxygen and thermal stability.

The main efforts were aimed at developing a procedure for depositing nanoparticles of platinum group metals (Ru, Rh, Pd and Pt) onto oxide supports, which would provide a high particle dispersion and adhesion to the support and be quite simple and adaptable for coating the structured substrates. 

As a result of the research, a method of sorption–hydrolytic precipitation was proposed [49]. The method is based on the slow kinetics of ligand exchange in alkaline solutions of chloride complexes of platinum metals. This approach allowed the selection of appropriate concentrations of metal chlorides and precipitant (Na_2_CO_3_) and a temperature to obtain a metastable solution, in which homogeneous precipitation of platinum metal hydroxides is impeded for kinetic reasons. After immersing the carrier into the solution, the precipitation of metal hydroxide particles in its pores proceeds by the heterogeneous nucleation mechanism.

**Figure 2:**
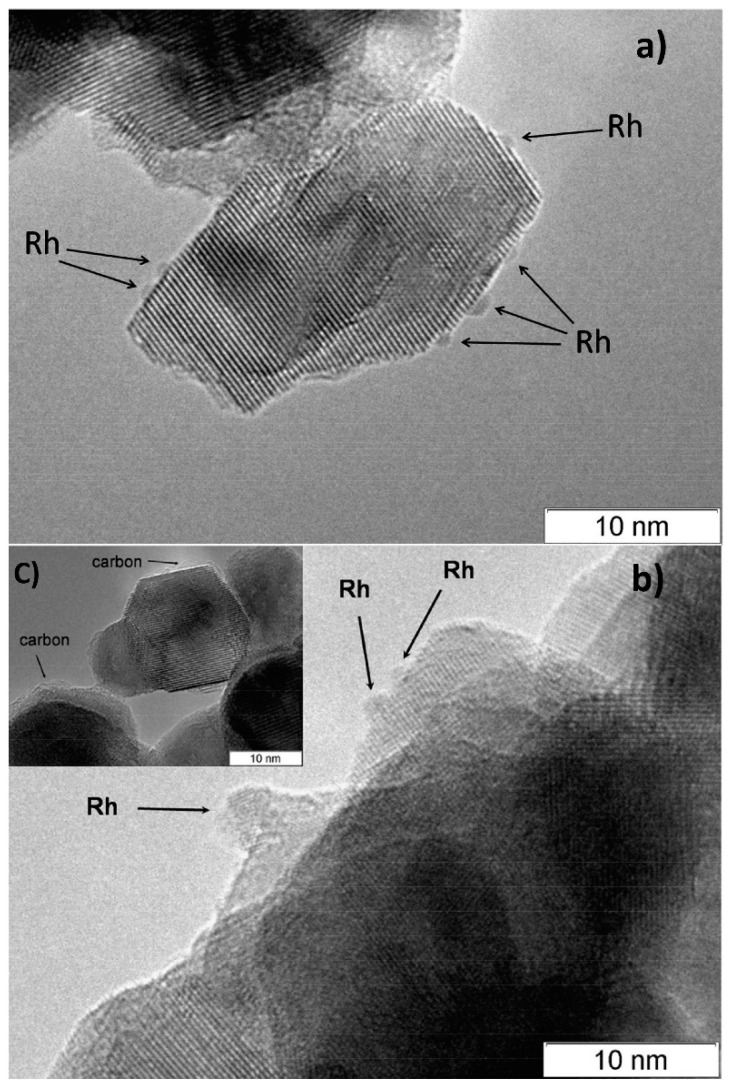
TEM images of 1 wt.% Rh/CZ: (**a**) as-prepared and (**b**,**c**) used in HD ATR [49].

According to CO chemisorption data, the average size of the Rh, Ru and Pt particles deposited by the sorption–hydrolytic procedure on a commercial support of composition Ce_0.75_Zr_0.25_O_2_ (hereinafter CZ) was 1.1, 1.2 and 1.8 nm, respectively [49]. Transmission electron microscopy (TEM) data showed that Rh particles exist on the support surface predominantly in the form of 1–2 nm clusters. (Figure 2a). After HD ATR experiments, the support crystallites increased in size to 20–30 nm, while the aggregate size remained the same (Figure 2b). Additionally, carbon species in the form of 1–2 graphite layers appeared occasionally on the surface (Figure 2c). The size of Rh particles increased slightly to 2–4 nm. After oxidative treatment of the catalyst, the carbon deposits disappeared, while the size of support crystallites and Rh particles remained unchanged.

Note that the DF ATR catalysts must be highly active and stable under DF SR conditions, since oxygen is rapidly consumed under ATR conditions and, in fact, most of the catalyst layer operates under SR conditions. The resulting Rh/CZ, Ru/CZ and Pt/CZ catalysts were studied in SR of n-hexadecane (HD), which served as a model DF compound. The fuel conversion was evaluated gravimetrically by collecting unreacted fuel into a condensate vessel and calculated using the following equation:

$$X\;(\%)=\frac{V_{0}*t-m}{V_{0}*t}*100,$$

where X (%) is fuel conversion, *V_0_*—fuel flow rate (g/h), *t*—sampling time (h) and *m*—sample weight (g).

**Figure 3:**
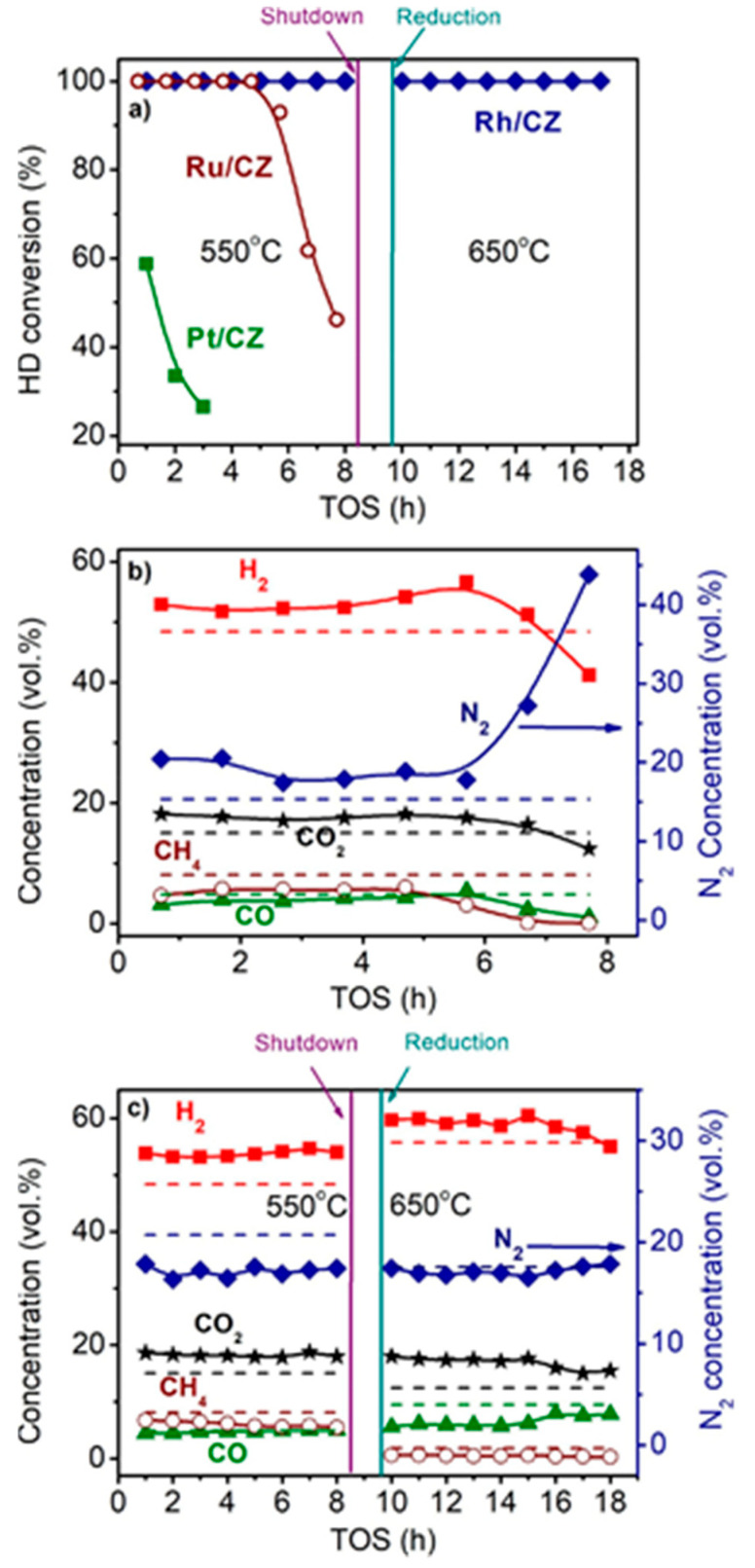
The HD conversion (**a**) and product distribution on dry basis (**b**,**c**) over 1.9 wt.% Pt/CZ (**a**), 1 wt.% Ru/CZ (**a**,**b**) and 1 wt.% Rh/CZ (**a**,**c**) in the HD SR as a function of time on stream at H_2_O/C = 3.0, T = 550–650 °C and GHSV = 23,000 h^−1^. Points—experiment, dashed line—equilibrium [49].

Figure 3 shows the time dependences of HD conversion, and product distribution for the Pt/CZ, Ru/CZ and Rh/CZ catalysts at HD SR. It is seen that Pt/CZ showed the worst catalytic properties: at 550 °C, it failed to achieve complete HD conversion and demonstrated its decrease from 59 to 27% within 3 h. The Ru/CZ catalyst rapidly lost activity after 5 h on stream and respective HD conversion decreased to 46%. The Rh/CZ catalyst demonstrated stable operation at 550 °C for 8 h, provided 100% HD conversion and the product concentrations (vol.%) of 54 H_2_, 18 CO_2_, 5 CO and ~6% CH_4_, which were close to the thermodynamically equilibrium ones. Then the catalyst was regenerated with hydrogen and retested at 650 °C. The HD conversion was 100%. The reaction product distribution was similar to the thermodynamically equilibrium one calculated for a temperature of 650 °C.

Thus, the activity and stability of the prepared noble-metal-based catalysts decreased in the following order: Rh/CZ > Ru/CZ >> Pt/CZ [49]—in good agreement with the results of other studies discussed above.

Note that catalysts supported on mixed cerium–zirconium oxides are usually inappropriate for practical use in granular form owing to insufficient mechanical strength and poor formability. Therefore, the feasibility of using a mixed Al_2_O_3_-Ce_0.75_Zr_0.25_O_2_ oxide as a support (commercial product 50 wt.% Al_2_O_3_ and 50 wt.% Ce_0.75_Zr_0.25_O_2_, hereinafter CZA) and alumina as a binding agent was investigated. The alumina additive is also intended to improve the catalyst thermal stability.

**Figure 4:**
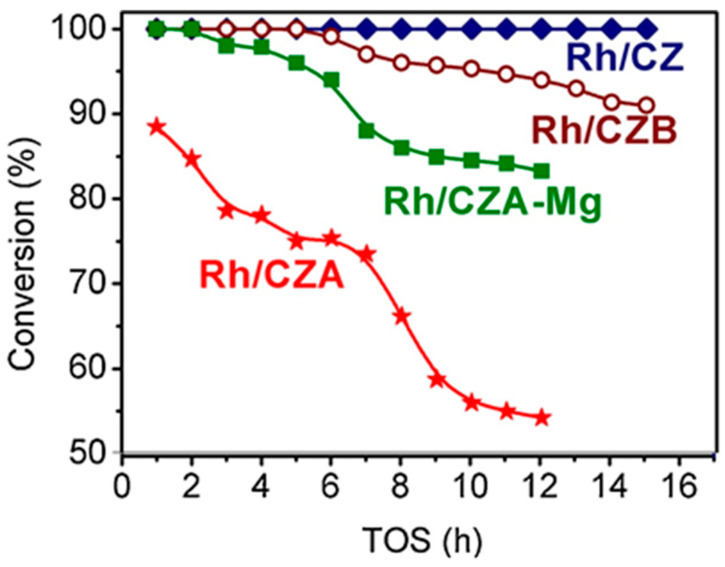
The time dependence of HD conversion under the following SR conditions: GHSV = 23,000 h^−1^, H_2_O/C = 3, T = 550 °C [50].

Besides Rh/CZ and Rh/CZA, Figure 4 presents the test results for Rh/CZA doped with MgO (Rh/CZA-Mg) and Rh-based catalyst supported on CZ containing 20 wt.% pseudoboehmite (Rh/CZB) as a structural promoter. Clearly, the Rh/CZ and Rh/CZB catalysts had the highest activity and coking resistance. The higher the aluminum content in the catalysts, the more rapidly they lost activity owing to acceleration of the side process of carbon deposition. Most likely, the catalyst deactivation is associated with the presence of acid sites on the alumina surface, while support doping with basic oxide MgO facilitated a significant increase in catalyst stability.

**Figure 5:**
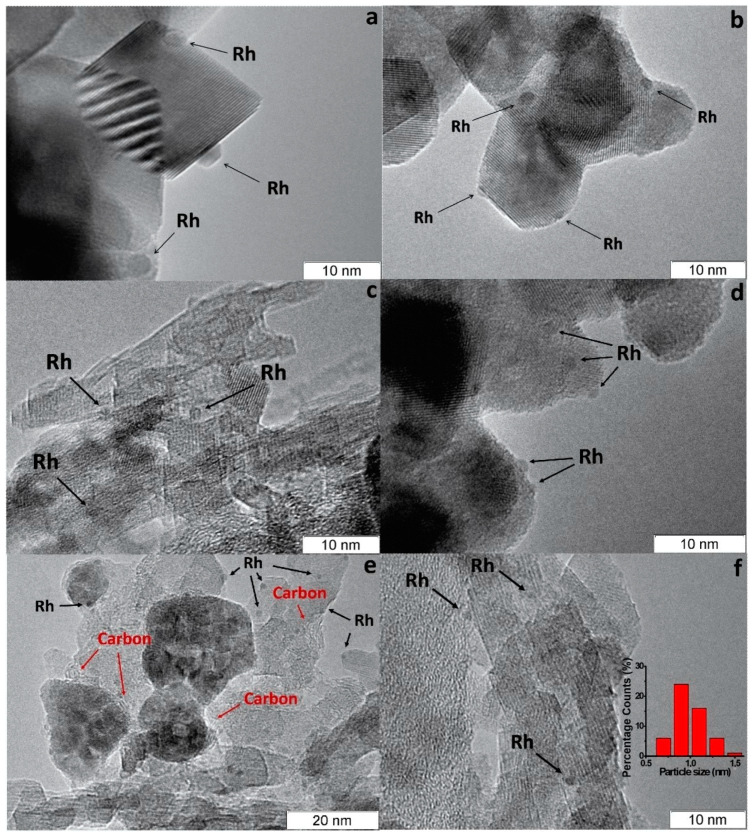
TEM images of as-prepared Rh/CZ (**a**), Rh/CZB (**c**) and used in HD SR Rh/CZ (**b**), Rh/CZB (**d**), Rh/CZA (**e**) and annealed Rh/CZA (**f**) catalysts [50].

Rh/CZA was the most susceptible to coking: it accumulated 3.7 wt.% of carbon in 12 h of HD SR. Since the size of Rh particles in Rh/CZA after annealing remained unchanged (Figure 5f), rapid deactivation of the catalyst is explained by coke formation. As known, γ-Al_2_O_3_ contains acid sites, which are responsible for coke formation [13,27]. As proved by the results of Rh/CZA-Mg catalytic activity tests (Figure 4) and TPO data [50], the blocking of acid sites by Mg cations causes a threefold decrease in the amount of carbon formed. Compared to Rh/CZA-Mg and Rh/CZA, the use of 20 wt.% pseudoboehmite as a binder in Rh/CZB improved the catalyst performance in HD SR (Figure 4), but the carbon productivity exceeded that of Rh/CZA-Mg. The same amount of carbon was observed in Rh/CZB after HD ATR. Among the studied catalysts, Rh/CZ accumulated the lowest amount of carbon: 1.2 wt.% for 15 h of HD SR and 1.5 wt.% for 12 h of HD ATR. 

Thus, to ensure the stable operation in DF SR and ATR of Rh-based catalysts supported on alumina-containing carriers, it is necessary to “neutralize” completely the acidity of the alumina surface [50].

## 3. Structured Composite Catalysts Supported on FeCrAl Alloy Wire Mesh

The DF ATR process is characterized by a combination of exo- and endothermic reactions. As discussed below in Section 8, modern understanding of the process mechanism assumes that fast complete oxidation reactions occur in the frontal part of the reactor with the release of heat, which is then consumed along the catalyst bed during the endothermic processes of steam and carbon-dioxide reforming of hydrocarbons. Therefore, when carrying out the ATR process, to prevent overheating in the frontal section and overcooling in the tail section, the structure of the catalyst bed must ensure efficient heat transfer between these zones. Conventional granular ceramic catalysts have low thermal conductivity and are hardly appropriate for this purpose. Besides, to reduce the pressure drop in the reactor, it is necessary to use large-size catalyst grains (1 cm and larger), which inevitably leads to a low utilization factor of the catalyst grain under conditions of fast ATR reactions.

For ensuring efficient implementation of the ATR process, it was proposed to use composite catalytic systems of the “metal nanoparticles/active oxide nanoparticles/structural oxide component/structured metal substrate” type. A structured metal substrate made of heat-resistant FeCrAl alloy facilitates fast heat removal/supply for exo-/endothermic reactions, has sufficient hydrodynamic characteristics, allows manufacturing products of various geometric shape and easy process scaling. The structural oxide component (alumina) provides thermal stability and high specific surface area and increases mechanical strength for the supported catalytic coating. The active oxide component (cerium oxide and mixed cerium–zirconium oxides with a fluorite structure) participates in the activation of water and oxygen molecules, improves the coke resistance because of high oxygen mobility and keeps the active component in a fine dispersed state owing to strong metal–carrier interaction. Metal nanoparticles of 1–2 nm size are involved in the activation of hydrocarbon molecules.

**Figure 6:**
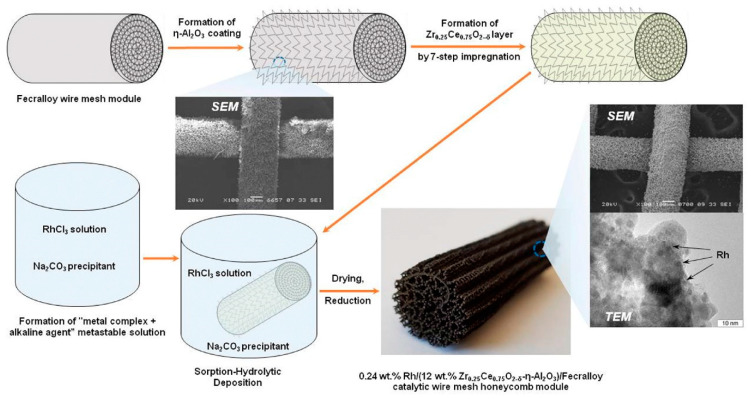
Schematic preparation procedure of Rh/CZB/FCA catalytic block, its general view, SEM and TEM images [51].

Based on this concept, the structured catalysts of composition 0.24 wt.% Rh/Ce_0.75_Zr_0.25_O_2_/Al_2_O_3_/FeCrAl (Rh/CZB/FCA) were prepared and tested. The structured metal substrate was made of FeCrAl alloy wire mesh (wire thickness of 0.25 mm, cell size of 0.5 × 0.5 mm). Structural oxide component, a layer of ƞ-Al_2_O_3_ in the amount of 6 wt.%, was supported on the metal substrate to ensure reliable adhesion of CZ active oxide nanoparticles (Figure 6). By calcining in air, α-Al_2_O_3_ layer was pre-formed on FeCrAl wire mesh; then a modified Bayer method (using aluminum hydroxide) was used to deposit an η-Al_2_O_3_ coating with a flexible (“breathing”) needle-shaped morphology [90]. According to SEM data (Figure 6), aluminum oxide consisted of tubular or acicular crystals (5–10 µm thick, up to 50 µm long).

The obtained ƞ-Al_2_O_3_/FeCrAl sample was repeatedly impregnated with a solution of Ce(NO_3_)_3_·6H_2_O and ZrO(NO_3_)_2_·2H_2_O (Ce/Zr = 3) and calcined at 800 °C [51]. Thus, the 12 wt.% Ce_0.75_Zr_0.25_O_2_/Al_2_O_3_/FeCrAl (CZB/FCA) composite support was obtained. Rh nanoparticles in the amount of 0.24 wt.% were deposited on the CZB/FCA by sorption–hydrolytic precipitation method. The RhCl_3_ solution was mixed with Na_2_CO_3_ in the ratio Na/Cl = 1. At room temperature, the resulting solution is metastable with regard to the homogeneous precipitation of rhodium hydroxide. The solution was brought into contact with CZB/FCA at T = 75 °C to initiate the hydrolysis that facilitated uniform deposition of rhodium particles throughout the structured support surface. At the final stage, the structured Rh/CZB/FCA catalyst was dried in air and reduced in hydrogen flow at 250 °C for 30 min.

**Figure 7:**
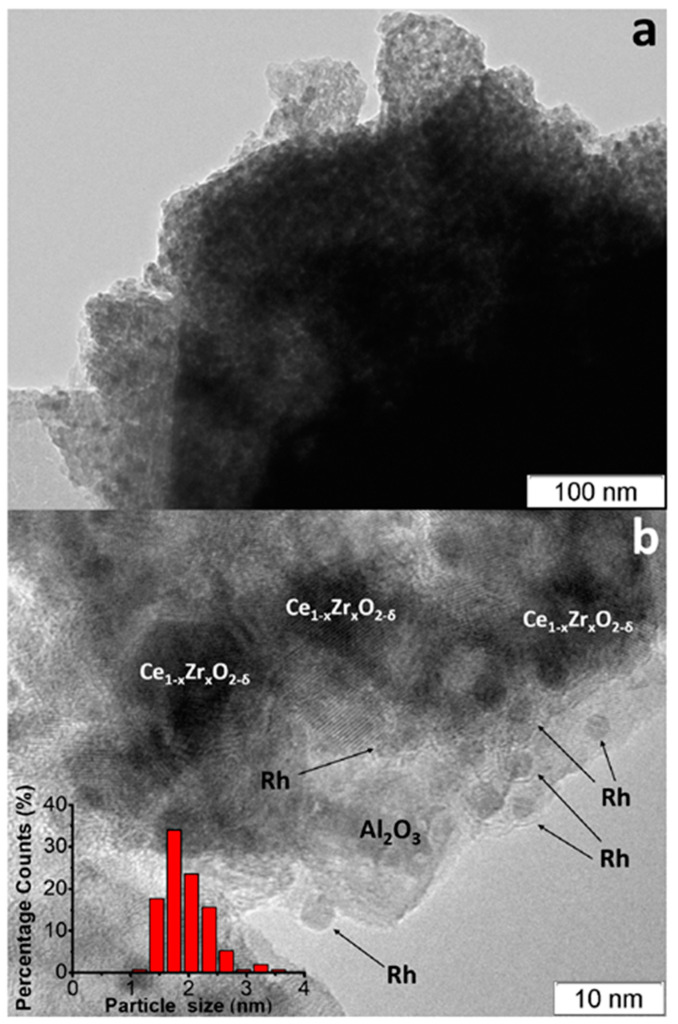
TEM images of as-prepared (**a**,**b**) Rh/Ce_0.75_Zr_0.25_O_2_-Al_2_O_3_/FeCrAl and Rh particles size distribution (**b**) [50].

The TEM images of as-prepared Rh/CZB/FCA catalyst (Figure 7) [50] show that the support consisted of ~1 µm-sized Al_2_O_3_ particles containing 5–20 nm Ce_1−x_Zr_x_O_2−δ_ crystallites on their surface and aggregated into large porous species. Rh particles on the support surface were predominantly in the form of 1–2 nm clusters, though 3–4 nm particles were observed as well (Figure 7b).

**Figure 8:**
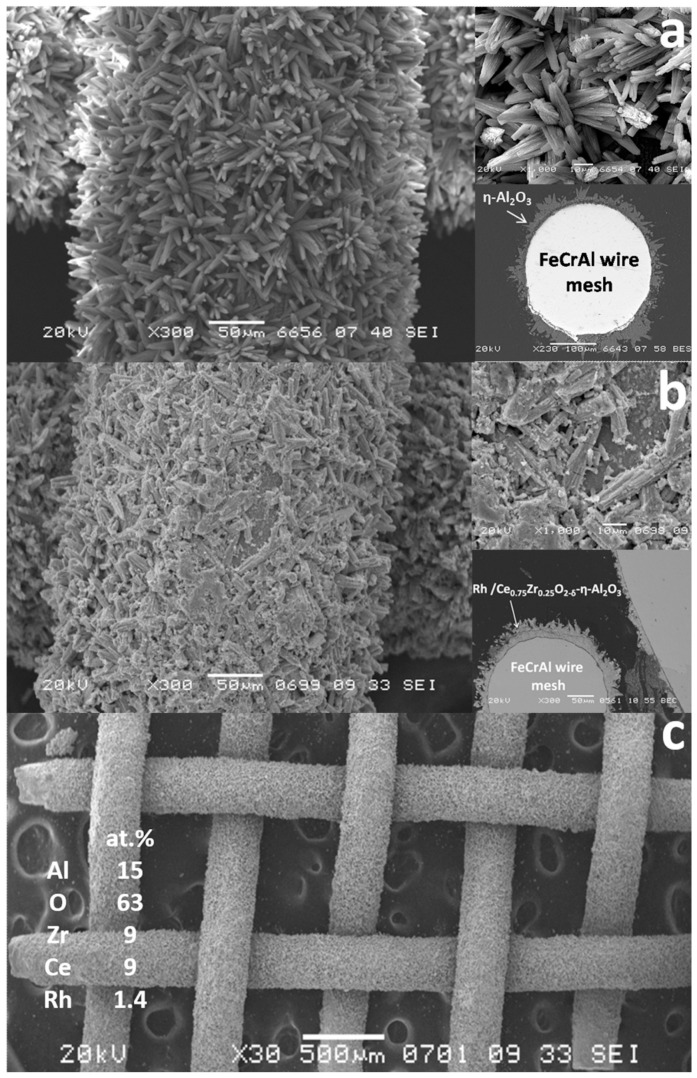
SEM images of ƞ-Al_2_O_3_/FeCrAl (**a**) and as-prepared Rh/Ce_0.75_Zr_0.25_O_2_-Al_2_O_3_/FeCrAl (**b**,**c**) catalyst [50].

The SEM micrographs of Al_2_O_3_/FeCrAl (Figure 8a) and Rh/CZB/FCA (Figure 8b,c) clearly show that the ƞ-Al_2_O_3_ layer consisting of tubular or acicular crystals (5–10 µm thick, up to 50 µm long) evenly covers the surface of the FeCrAl wire mesh. The thickness of the ƞ-Al_2_O_3_ layer was 30–50 μm. After depositing Rh/Ce_1−x_Zr_x_O_2−δ_ onto the ƞ-Al_2_O_3_ layer, the surface microstructure changed (Figure 8b): the crystal surface became rougher, though the thickness of the final layer remained the same (30–50 µm).

According to EDX data for several 100 × 100 µm areas, the surface of the Rh/CZB/FCA catalyst contained Rh, Ce, Zr, Al and O. The element concentrations in all regions were the same, which proves a uniform distribution of Rh and Ce_1−x_Zr_x_O_2−δ_ over the surface of alumina crystals and agrees well with the TEM data.

Thus, it was shown [50] that the structured catalyst Rh/Ce_0.75_Zr_0.25_O_2_-Al_2_O_3_/FeCrAl is a composite in which aluminum oxide, chemically bonded to the metal substrate, provides the mechanical strength of the catalytic layer and keeps Rh and Ce_1−x_Zr_x_O_2−δ_ particles in a highly dispersed state.

**Figure 9:**
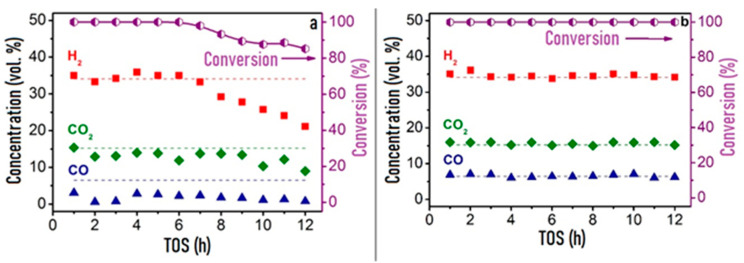
The time-on-steam dependence of HD conversion under ATR conditions: WHSV 30,000 cm^3^g^−1^h^−1^ (**a**) and 90,000 cm^3^g^−1^h^−1^ (**b**), T = 650 °C, H_2_O/C = 2.5, O_2_/C= 0.5 over Rh/CZB (**a**) and Rh/CZB/FCA (**b**) catalysts.Adapted from [50].

In comparative studies of the catalytic properties of granular Rh/CZB and composite Rh/CZB/FCA in HD ATR, the Rh/CZB catalyst showed stable operation for 6 h on stream and product distribution close to the thermodynamically equilibrium one. Furthermore, the HD conversion and concentrations of the main products decreased, while the outlet concentrations of C_2_–C_5_ components increased (Figure 9a). Rh/CZB/FCA under HD ATR conditions demonstrated a 100% HD conversion for 12 h on stream (Figure 9b) even at a higher space velocity compared to that in the experiment with the granular catalyst. The outlet product concentrations were close to the thermodynamically equilibrium values. Thus, the structured Rh/CZB/FCA catalyst showed high activity in HD ATR and provided hydrogen productivity of 2.5 kg_H_2__kg_cat_^−1^h^−1^; it obviously possesses a high potential for the ATR of commercial diesel fuel.

## 4. Testing of Structured Catalysts

Catalytic modules of the same composition (Rh/Ce_0.75_Zr_0.25_O_2−_-Al_2_O_3_/FeCrAl), but different lengths—10, 20 and 60 mm (hereinafter referred to as Rh10, Rh20 and Rh60, respectively)—were used in several series of experiments on HD SR and HD ATR to obtain detailed information on the outlet product distribution and temperature profile along the length of the catalytic block [96]. All tests were carried out at constant temperature (T = 750 °C) and inlet molar ratios H_2_O/C = 2.6 and O_2_/C = 0.4 and with variable space velocity of the reaction mixture.

It was found [96] that the HD conversion in SR experiments increased with the length of the catalytic block and amounted to 77, 80 and 92% for Rh10, Rh20 and Rh60, respectively; the outlet concentrations of H_2_, CO and CO_2_ increased as well and approached thermodynamically equilibrium values for the Rh60 catalyst. The outlet C_1_–C_5_ byproduct concentrations decreased (especially in the case of ethylene—from 7.5 to ~2 vol.%) with increasing catalytic block length. 

The HD conversion byproducts (C_2_–C_5_ hydrocarbons) mainly contained 1-alkenes. Based on the product distribution data, it was assumed that during the SR process, thermal cracking of HD takes place to form 1-alkenes and hydrogen; CO and CO_2_ are formed by the reaction of steam with carbon-containing intermediates on the catalyst surface. It also cannot be excluded that the hydrogen released in these processes participates in the hydrocracking reaction with the formation of light alkanes.

Another series of experiments with catalytic blocks of different lengths was performed under HD ATR conditions [96]. It demonstrated significantly different data on the HD conversion, target products distribution and composition of byproducts and intermediates (C_2_–C_5_ hydrocarbons), compared to respective HD SR data. 

It should be noted that none of the experiments demonstrated unreacted oxygen at the reactor outlet. HD ATR in the presence of oxygen and steam proceeds very quickly: the required contact time of the reaction mixture with the catalyst did not exceed 0.03 s (i.e., even a 10 mm long catalytic block was sufficient). A sixfold longer contact time—0.18 s (block of 60 mm length)—was needed only to reach thermodynamic equilibrium between the target reaction products (H_2_, CO and CO_2_), and to reduce the content of reaction byproducts (primarily ethylene). For Rh60, the outlet concentrations of CH_4_, C_2_H_4_, C_3_H_6_ and C_4_H_8_ were 200, 400, 90 and 20 ppm, respectively.

**Figure 10:**
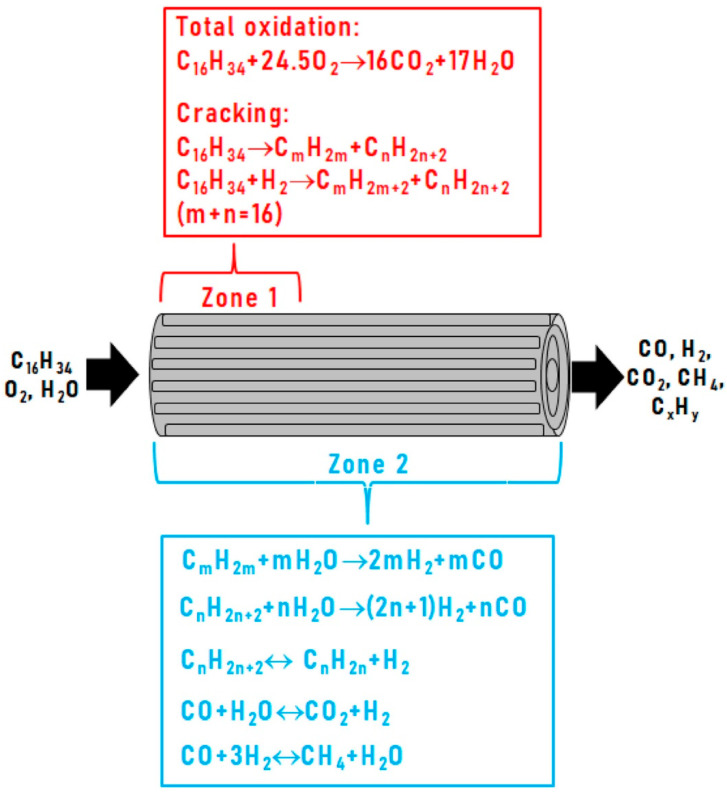
Scheme of autothermal reforming of hexadecane [96].

Based on the results obtained, the catalytic block under HD ATR conditions can be conditionally divided into two zones (Figure 10): zone 1, where fast reactions of deep oxidation and cracking occur, and zone 2, which involves the slower processes of steam reforming, dehydrogenation and methanation, proceeding along the entire length of the catalytic layer and being responsible for the formation of the final reaction products [96].

**Figure 11:**
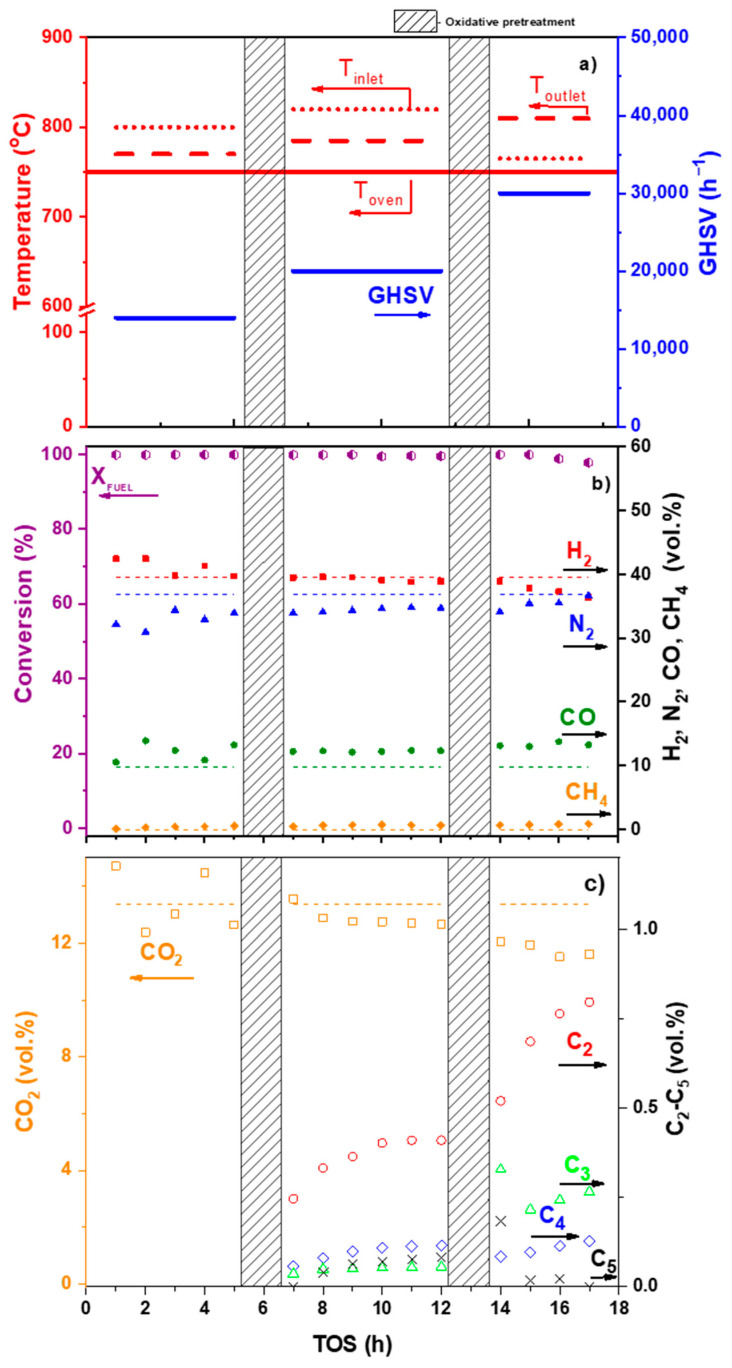
DF ATR over Rh/CZB/FCA under the following conditions: GHSV 14,000–30,000 h^−1^, T_oven_ = 750 °C, H_2_O/C = 2.5, O_2_/C = 0.4. Experimental conditions (**a**); diesel fuel conversion (%) and H_2_, N_2_, CO, CH_4_ concentrations (vol. %) (dry basis) (**b**); CO _2_ and by-products concentrations (vol. %) (dry basis) (**c**). Points—experiment; dashed lines—equilibrium concentrations. Adapted from [91].

The studies of the DF ATR process (Figure 11) revealed that at a temperature of 750 °C and a space velocity of 30,000 h^−1^, the DF conversion decreased from 100 to 97.8% in 4 h and the catalyst suffered coking. The oily residue collected from the surface of the aqueous condensate at the reactor outlet contained 78 wt.% of mono-, di- and polyaromatic hydrocarbons (Table 2), which are hardly convertible to synthesis gas and, most likely, contribute to the formation of carbon on the catalyst surface.

To check the assumption about the key effect of di- and polyaromatic compounds on the fuel reforming process, comparative experiments were carried out using catalytic blocks of different lengths in the ATR of DF model blend containing various classes of organic compounds: 75 wt.% hexadecane + 20 wt.% o-xylene + 5 wt.% naphthalene [97].

**Figure 12:**
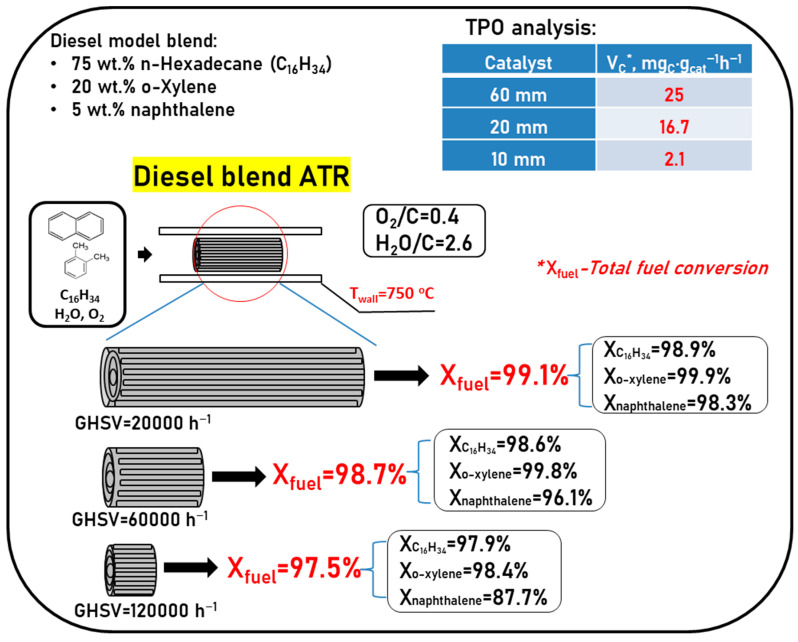
Autothermal reforming of DF model blend over Rh10, Rh20 and Rh60 at furnace temperature 750 °C. Adapted from [97].

Conversion of hydrocarbons at ATR of the DF model blend increased with the increasing lengths of the catalytic blocks (decreasing GHSV) as follows: 97.5% for Rh10, 98.7% for Rh20 and 99.1% for Rh60. As Figure 12 shows, the lower the GHSV, the higher the conversion of both aliphatic and mono/diaromatic hydrocarbons. However, in the presence of aromatic compounds, the conversion of n-hexadecane decreases considerably (without aromatics, the HD conversion exceeds 99% even on Rh 10). The set of organic compounds in the oily residue was identified by GC-MS analysis. These compounds represent byproducts and intermediates of the reactions of alkylation, dealkylation, isomerization, condensation, cracking and dehydrogenation accompanying the ATR process [97].

TPO experiments show (Figure 12) that the most profound increase (around an order of magnitude) of the average specific velocity of carbon formation is observed when changing from module Rh 10 to Rh 20, and less significant—between modules Rh20 and Rh 60. These data mean that the reaction of complete oxidation, which proceeds in the frontal catalyst section at the higher temperature, prevents carbon accumulation even in the presence of large amounts of aromatic hydrocarbons in the feed mixture. In the next catalyst sections, where the endothermic reactions of hydrocarbon conversion proceed and the temperature becomes lower, considerable catalyst coking is observed. Carbon formation is promoted both by the condensation reactions of low reactive aromatic compounds and by high gas-phase concentration of ethylene—a known precursor of carbon deposits on the catalyst surface [97].

**Figure 13:**
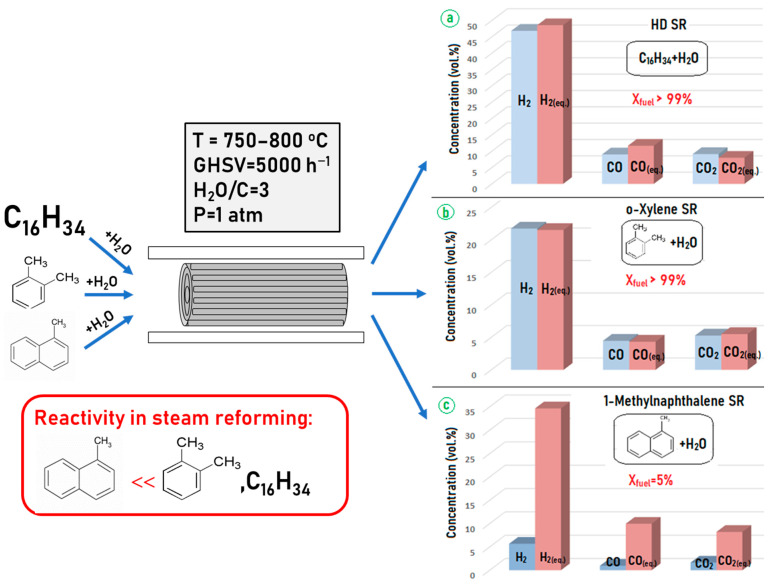
Steam reforming of HD (**a**), o-xylene (**b**) and 1-methylnaphthalene (**c**) over Rh60. Comparison of products concentrations with equilibrium values (red columns). Adapted from [97].

As mentioned above, all oxygen at diesel fuel ATR is consumed in the narrow catalyst layer at the frontal section of the block. Most likely, the reaction of complete oxidation involves predominantly aliphatic hydrocarbons that have a lower C–C bond energy. Aromatic compounds react with oxygen much more slowly owing to their lower activity caused by strong carbon–carbon bonds in the aromatic ring. To compare the reactivity of aliphatic, mono- and diaromatic hydrocarbons under steam reforming conditions, a set of experiments with individual compounds—HD, o-xylene and 1-methylnaphthalene—were performed using catalytic block Rh60 (Figure 13).

It is seen that the Rh60 catalyst is efficient in the SR of aliphatic and monoaromatic compounds, but demonstrates a relatively low efficiency in the reforming of di- and polyaromatic compounds. In fact, the situation occurs when aliphatic hydrocarbons are converted in the frontal part of the catalytic block at high temperature, whereas less reactive compounds undergo SR in the tail part of the block in the presence of synthesis gas components. Therefore, the key factor for ensuring stable operation of DF ATR catalysts is their activity and stability in SR of di- and polyaromatic compounds [97].

## 5. Catalyst Coking and Regeneration

To study the process of catalyst coking and predominant carbon localization in the catalyst structure, a series of experiments was carried out with a sample of composition 0.24 wt.% Rh/6%Ce_0.75_Zr_0.25_O_2_/6%Al_2_O_3_/FeCrAl.

**Figure 14:**
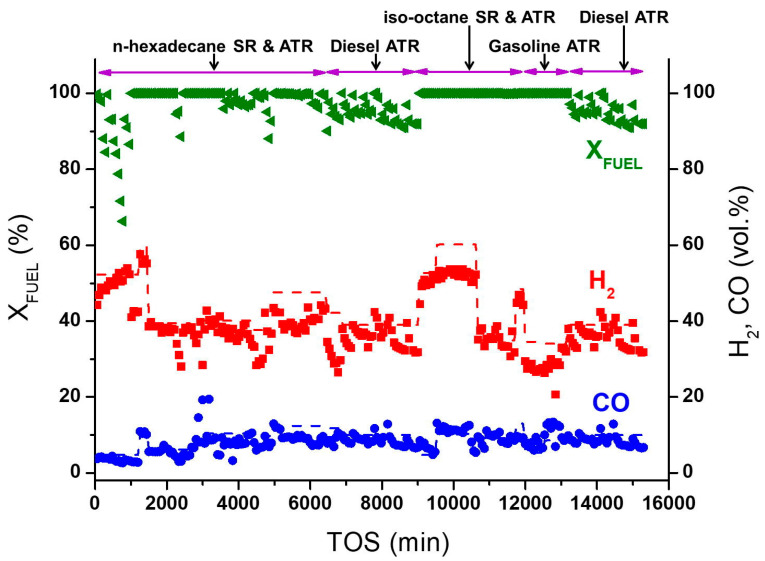
Fuel conversion (X_FUEL_), H_2_ and CO concentrations (dry basis) as a function of time on stream during SR and ATR of different fuels. Points—experimental data, dashed lines—equilibrium concentrations [93].

The catalytic block was tested in the SR and ATR of various hydrocarbon fuels in a quartz fixed-bed flow reactor for about 250 h (Figure 14) under the following operating conditions: atmospheric pressure, temperature range 550–800 °C, GHSV 1000–20,000 h^−1^, molar ratios H_2_O/C = 2–3, O_2_/C = 0.28–0.5. The experiment was stopped when the catalyst turned to a deactivated state after DF ATP. The number of regeneration procedures in air flow at 600 °C exceeded 30 [93].

After the experiments, various parts of the catalytic block were examined. The outer layer of the catalyst was removed mechanically, the cylindrical structure was untwisted, and the sections located in the front and end zones of the catalytic block were cut out from the mesh for SEM analysis. Besides, a part of the catalytic coating (from the front and end zones of the catalytic block) was removed from the surface of the wire mesh and examined by TEM method. For comparison, the samples from the front and end parts of the as-prepared catalytic block (reference sample) were examined using the same methods. The samples for analysis were collected in this manner because autothermal reforming combines exothermic total oxidation reactions and endothermic reactions of steam reforming. As noted earlier, the process of complete oxidation of hydrocarbons proceeds quickly and is localized in the front part of the catalytic block, while steam reforming is a slower process and covers almost the entire catalytic block length [93].

**Figure 15:**
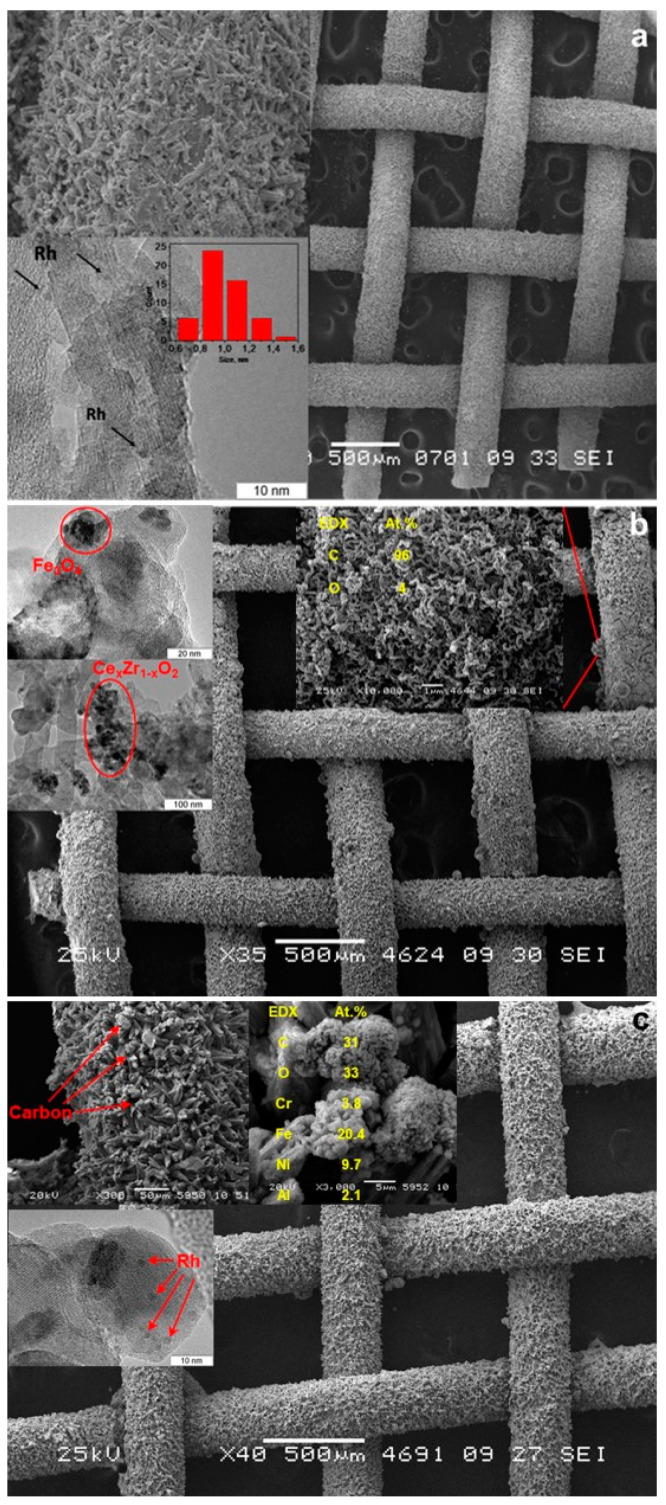
The TEM and SEM images and respective EDX patterns for the as-prepared (**a**) and the used catalytic block (250 h on stream): the frontal part (**b**) and the end part (**c**) [93].

The SEM images (Figure 15) of the as-prepared and used catalytic blocks showed that the catalytic coating stayed undamaged (kept integrity) after SR and ATR of hydrocarbon fuels. The Rh/Ce_0.75_Zr_0.25_O_2_/Al_2_O_3_ coating remained dense and uniform both at the front and at the end parts of the catalytic block. This result is particularly significant for the front part of the catalytic block since it is subjected to rapid temperature fluctuations during the start-up and shutdown procedures, and exactly in this part, the highest temperature is reached during stationary operation.

The formation of carbon “knobs” 5–50 µm in size was observed on the surface of both the front and end parts of the block after 250 h on stream. According to TEM and EDX data, the “knobs” were formed from carbon nanofibers. The TEM and EDX analyses revealed also the presence of iron particles near to these carbon species. Probably, coke formation on the catalyst surface is promoted by nano-sized iron impurities. This assumption seems to be quite reasonable because iron is known as one of the best catalysts for the growth of carbon nanofibers and nanotubes. Iron on the catalyst surface can appear both from the FeCrAl structural support during high-temperature transformations and from the reactor material. Another potential source of iron is the thermocouple jacket material, which has contact with the catalytic block and undergoes slow corrosion in the presence of steam and oxygen at temperatures of 750–800 °C at the block inlet. The problem of how Fe nanoparticles appear on the catalyst surface requires further study. This example clearly shows that under extreme reaction conditions of DF ATR, undesirable side processes can be initiated by materials other than those of the structured catalyst. In this regard, the choice of appropriate materials for the manufacture of various parts of the reformer and auxiliary devices seems to be of key importance for providing long-term and stable catalyst operation.

The TEM images of the used catalytic block show that the Rh nanoparticles kept a highly dispersed state and shape. This observation proves the high stability of the catalyst microstructure, at least in the end part of the catalytic block. Unfortunately, attempts to get acceptable-quality images of Rh particles in the front part of the used catalytic block appeared unsuccessful.

Microscopic studies of the catalytic block confirmed the formation of carbon nanofibers on the catalyst surface at DF ATR. It should be noted that the process of coke formation occurs on the surface of the catalytic coating and causes no destruction of it. Most likely, the formation of carbon fibers is caused by contamination of the catalytic coating with iron nanoparticles. The location of carbon deposits on the catalyst surface allows their simple oxidation and removal during regeneration. Thus, it was shown that the prepared catalytic block is stable and can be regenerated under the conditions of SR and ATR of hydrocarbons; no morphological violations and microstructure degradations were observed in either the frontal or tail parts of the catalytic block.

Additionally, the processes of carbon removal from the catalyst surface in the process of oxidation by steam or oxygen were studied. For this purpose, the Rh/CZB/FCA catalyst was subjected to preliminary coking under the DF ATR conditions. The catalyst regeneration by oxygen or steam was performed in a flow of composition 20 vol.% O_2_ and 80 vol.% Ar or 75 vol.% H_2_O and 25 vol.% Ar, respectively, at the furnace heating rate of 10 °C/min from 350 °C to 750 °C. The product distribution was determined using a Stanford Research QMS 200 mass spectrometer in real time.

**Figure 16:**
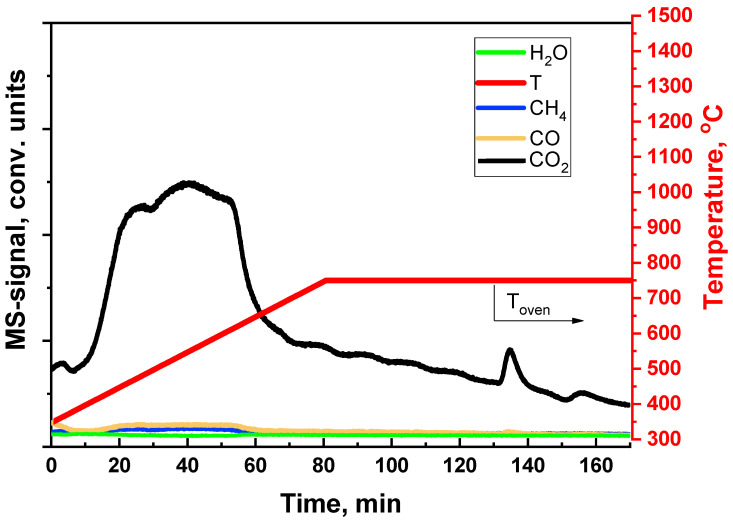
Removal of carbon, deposited on the Rh/CZB/FCA surface at DF ATR, by oxygen.

It was found (Figure 16) that the reaction of carbon deposit oxidation by oxygen begins to proceed actively at a temperature of 450 °C and releases a large amount of CO_2_. The main part of the carbon is oxidized long before 750 °C, which is the operating temperature at DF ATR. After the catalyst was regenerated, its activity in DF ATR turned to the initial level without degradation of the catalytic coating.

**Figure 17:**
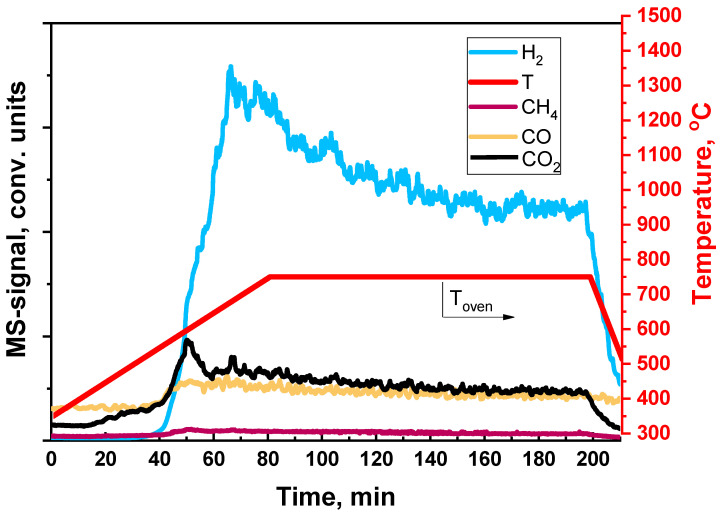
Removal of carbon, deposited on the Rh/CZB/FCA surface at DF ATR, by steam.

In experiments on catalyst regeneration by steam (Figure 17), the reaction of carbon deposit oxidation proceeds actively starting from the temperature of 550 °C and is accompanied by the formation of hydrogen (Figure 17). The high measured values of hydrogen concentration, in comparison with those of other reaction products, are associated with the high sensitivity of the device to hydrogen. Probably, H_2_ is formed not only by the whisker carbon oxidation reaction, but also by the steam reforming of gum carbon, which contains hydrogen in its composition. Traces of carbon were observed even after two hours of catalyst regeneration by steam at the furnace temperature of 750 °C.

The performed studies showed that oxidative regeneration with air is the most effective way to remove carbon deposited on the catalyst during DF ATR. At the same time, water vapor is also able to oxidize the carbon deposits, but at a lower rate. The rate of carbon deposit oxidation by steam at the DF ATR operating temperatures dictates the period of catalyst life between regenerations. This parameter is one of the most important properties of the catalyst: upon reaching a certain (perfect) rate, the need for regeneration can be avoided completely. Note that water vapor oxidizes carbon only at high temperatures; therefore, to ensure stable operation of the catalyst with real diesel fuel containing di- and polyaromatics, it is recommended to increase the temperature at the end part of the catalytic block. This purpose can be achieved, for example, by increasing the O_2_:C input ratio.

## 6. Testing of Diesel Reformer

**Figure 18:**
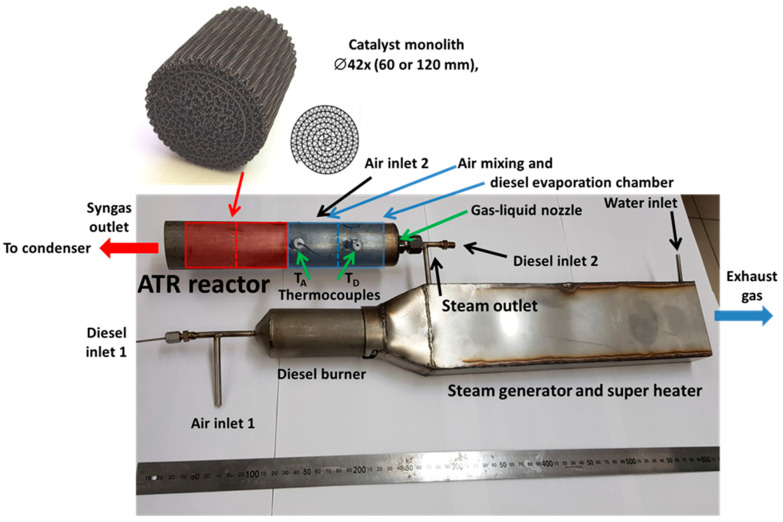
A general view of the ATR diesel reactor together with a starting diesel burner, an evaporator and a steam superheater [91].

To carry up-scale tests, a model diesel fuel reformer was developed, which included a diesel fuel burner, a steam generator and a superheater, a gas-liquid nozzle for DF evaporation by hot steam, a zone for mixing the steam-fuel blend with air and the 0.24 wt.% Rh/6%Ce_0.75_Zr_0.25_O_2_/6%Al_2_O_3_/FeCrAl-structured catalytic block with a diameter of 42 mm and a length of 120 mm (Figure 18).

**Figure 19:**
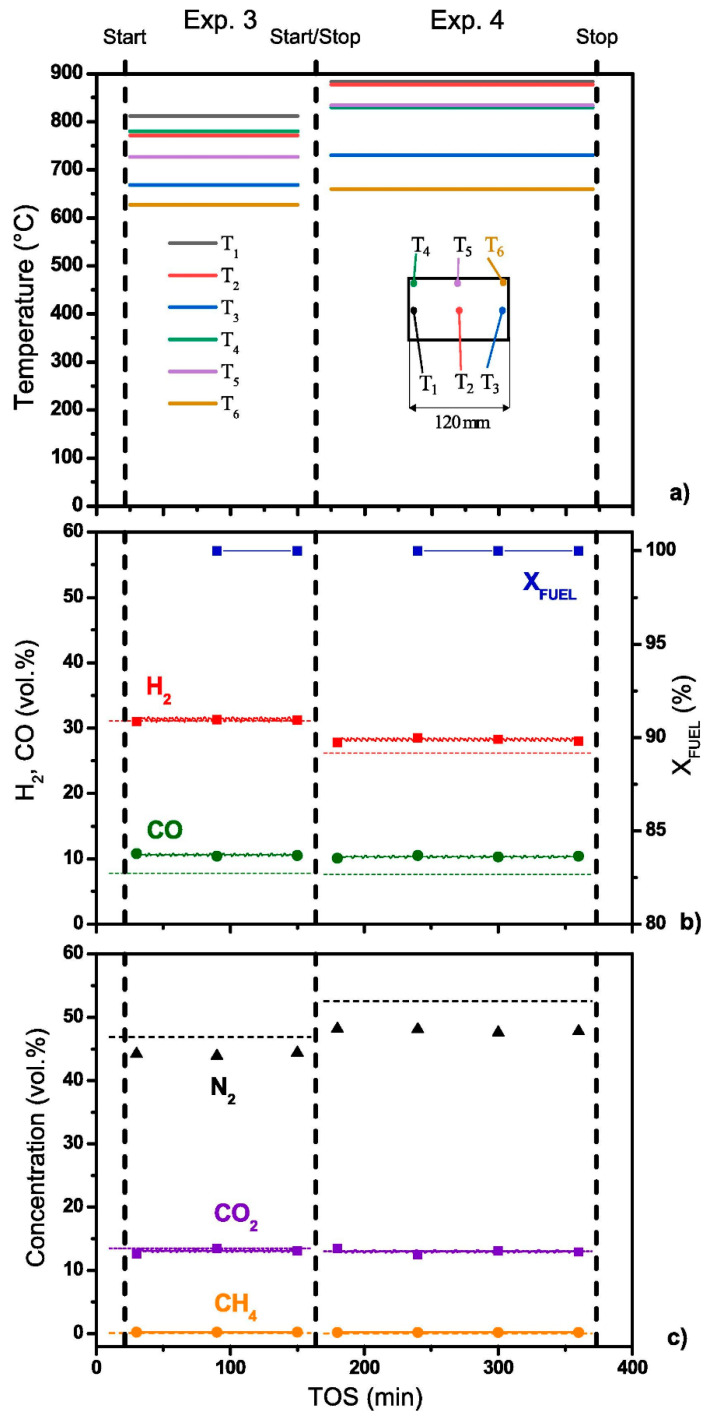
The temperatures in six points of Rh/CZ-42-120 catalytic block (**a**), diesel conversion, H_2_ and CO (**b**), N_2_, CO_2_ and CH_4_ concentrations (dry basis) (**c**) as a function of time on stream during DF ATR experiments. Points and solid lines—experimental data, dashed lines—equilibrium concentrations. Experimental conditions: O_2_:C = 0.6; GHSV = 6750 h^−1^; O_2_:C = 0.7, GHSV = 7500 h^−1^ [91].

The studies were carried out at high molar ratios O_2_/C = 0.6 and 0.7 to reduce the coking processes; the reaction mixture space velocity was 6750 and 7500 h^−1^, respectively. Six thermocouples were inserted into the catalytic block to record the temperature in certain points. Figure 19a demonstrates the thermocouple locations and a typical temperature profile inside the catalytic block during experiments after reaching a steady state. Clearly, the temperature in different parts of the catalytic block was constant in time.

Under these reaction conditions, the synthesis gas productivity of the reformer was about 0.5 m^3^/h. Figure 19b,c show the synthesis gas composition on a dry gas basis.

Despite the high total aromatics content in DF (Table 2), the condensate at the reformer outlet consisted of an almost clear aqueous solution with a faint odor. HPLC analysis revealed no noticeable amounts of unreacted hydrocarbons in the solution.

Thus, upscaling the catalyst and ATR process of diesel fuel with a high content of aromatic hydrocarbons was proved feasible. The performance of the model reformer is sufficient to feed a 0.5 kW high-temperature SOFC. The complete conversion of the di-aromatic components of diesel fuel is most likely associated with the high temperature of the block owing to using gas mixtures with a high O_2_:C molar ratio [91].

## 7. Development of a Mathematical Model for the Diesel Fuel Conversion

For further process upscaling, a mathematical model of the DF ATR over a 0.24 wt.% Rh/6%Ce_0.75_Zr_0.25_O_2_/6%Al_2_O_3_/FeCrAl-structured catalyst was developed. HD was used as a model fuel in the calculations. To build a stationary model using Comsol Multiphysics software (version 6.0), the catalytic block was represented by a homogeneous porous medium, and the computational domain was defined in a two-dimensional axisymmetric geometry [94,95,96,98].

For the modeling, a simplified set of reactions was used, which described the main stages of the reforming:

(4) $$C_{16}H_{34}+24.5O_{2}\to 16{\mathrm{CO}}_{2}+17H_{2}O\;\;\;\Delta H_{298}=-9800\;\mathrm{kJ}/\mathrm{mol}$$

(5) $$C_{16}H_{34}+16H_{2}O\to 33H_{2}+16\mathrm{CO}\;\;\;\;\Delta H_{298}=2500\;\mathrm{kJ}/\mathrm{mol}$$

(6) $$\mathrm{CO}+H_{2}O↔{\mathrm{CO}}_{2}+H_{2}\;\;\;\;\;\;\;\Delta H_{298}=-41\;\mathrm{kJ}/\mathrm{mol}$$

(7) $$\mathrm{CO}+3H_{2}↔{\mathrm{CH}}_{4}+H_{2}O\;\;\;\;\;\;\;\Delta H_{298}=-206\;\mathrm{kJ}/\mathrm{mol}$$

(8) $$\mathrm{CO}+0.5O_{2}\;\to {\mathrm{CO}}_{2}\;\;\;\;\;\;\;\;\;\Delta H_{298}=-283\;\mathrm{kJ}/\mathrm{mol}$$

(9) $$H_{2}+0.5O_{2}\;\to H_{2}O\;\;\;\;\;\;\;\;\;\;\Delta H_{298}=-286\;\mathrm{kJ}/\mathrm{mol}$$

Reactions were also introduced in the model to account for the formation of light hydrocarbons, namely, alkanes and alkenes C_2_–C_5_. Since most of them are represented by C_2_ hydrocarbons, only ethane and ethylene were considered in the model. Possible routes for the formation of C_2+_ compounds are the hydrogenolysis of hexadecane and the interaction of CO with hydrogen, similar to the methanation Reaction (7). Thermodynamic analysis showed that the first route is the most probable, so the final set of assumed reactions for the formation and transformation of C_2+_ compounds was represented by the following reactions:

(10) $$C_{16}H_{34}+7H_{2}\to 8C_{2}H_{6}\;\;\;\;\;\;\;\;\;\Delta H_{298}=-305\;\mathrm{kJ}/\mathrm{mol}$$

(11) $$C_{2}H_{6}↔C_{2}H_{4}+H_{2}\;\;\;\;\;\;\;\;\;\Delta H_{298}=137\;\mathrm{kJ}/\mathrm{mol}$$

(12) $$C_{2}H_{6}+H_{2}\to 2{\mathrm{CH}}_{4}\;\;\;\;\;\;\;\;\;\Delta H_{298}=-65\;\mathrm{kJ}/\mathrm{mol}$$

(13) $$C_{2}H_{6}+2H_{2}O\to 5H_{2}+2\mathrm{CO}\;\;\;\;\;\;\;\Delta H_{298}=348\;\mathrm{kJ}/\mathrm{mol}$$

(14) $$C_{2}H_{4}+2H_{2}O\to 4H_{2}+2\mathrm{CO}\;\;\;\;\;\;\;\Delta H_{298}=315\;\mathrm{kJ}/\mathrm{mol}$$

(15) $$C_{2}H_{6}+3.5O_{2}\to 2{\mathrm{CO}}_{2}+3H_{2}O\;\;\;\;\;\;\Delta H_{298}=-1428\;\mathrm{kJ}/\mathrm{mol}$$

(16) $$C_{2}H_{4}+3O_{2}\to 2{\mathrm{CO}}_{2}+2H_{2}O\;\;\;\;\;\;\Delta H_{298}=-1323\;\mathrm{kJ}/\mathrm{mol}$$

In real conditions, Reactions (4), (5) and (10) proceed through several stages with successive decomposition of HD and the formation of various intermediates. However, none of the intermediates were found in the outlet gas mixture; therefore, the presented total process scheme was used.

Another important issue is related to the probable cracking of hexadecane without the participation of hydrogen. However, when the reaction of this type was added to the scheme, no improvement in the description of the experimental data was reached, and it was decided to exclude this stage from consideration.

The mathematical model included the processes of mass transfer and heat transfer, accounting for the changes in the velocity and pressure fields in the system [94,95].

**Figure 20:**
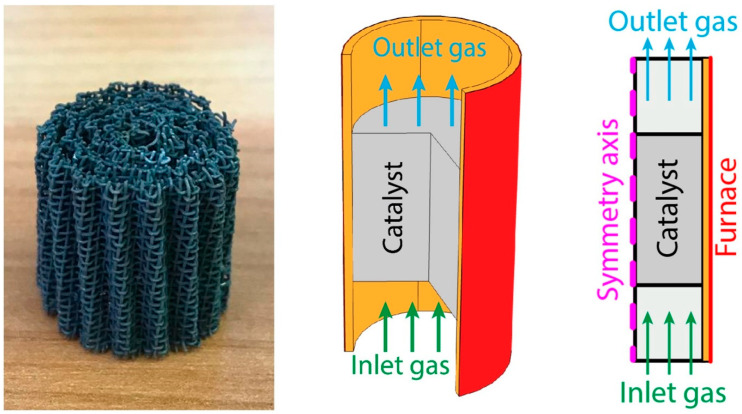
The catalytic block (**left**), considered 3D process geometry (**center**) and 2D axisymmetric computational domain (**right**) [95].

When solving the minimization task for various operating temperatures, the following set of kinetic equations and kinetic parameter values was obtained that provided the best matching between the experimental and calculated data (Table 3):

At mathematical modeling, the 3D geometry was simplified to a two-dimensional axisymmetric one assuming a homogeneous porous medium in the catalytic block region (Figure 20). The geometry included also the region of the gas flow (10 mm long) before and after the catalytic block (cylinder with a radius of 9 mm and a length of 20 mm) with insulating material (a layer of 1 mm thick mineral wool along the entire outer surface—orange area in Figure 20) [95].

The inlet temperature of the gas flow was set according to the experimental readings of a thermocouple located before the catalytic block inlet. The temperature at the outer surface of the insulating material was set equal to the furnace temperature. An infinitely fast heat exchange was set at the boundary between the catalyst and mineral wool, i.e., the temperatures of both sides were equal.

**Figure 21:**
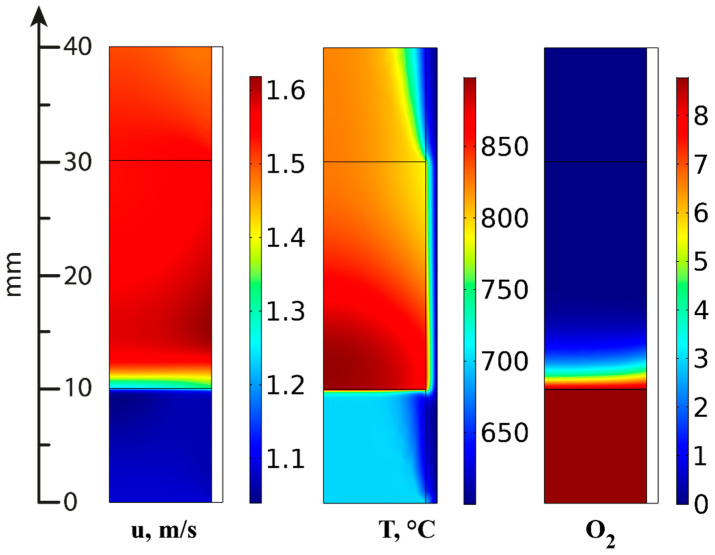
Gas velocity (**left**), temperature distribution (**center**) and oxygen concentration (**right**; vol.%) in ATR mode [95].

**Figure 22:**
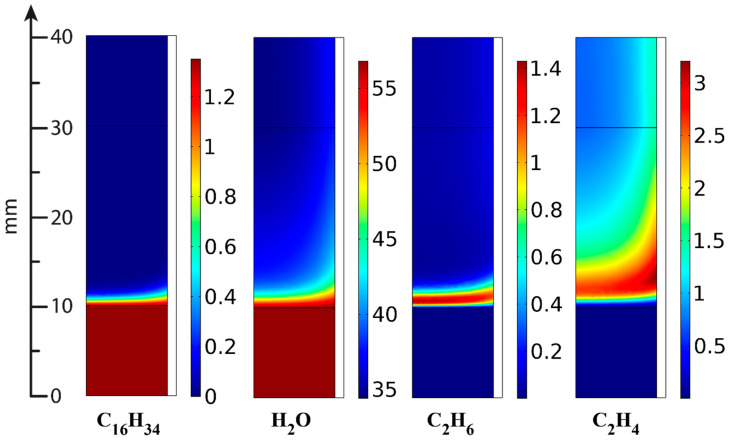
C_16_H_34_, water, C_2_H_6_ and C_2_H_4_ concentrations (vol.%) distribution in ATR [95].

**Figure 23:**
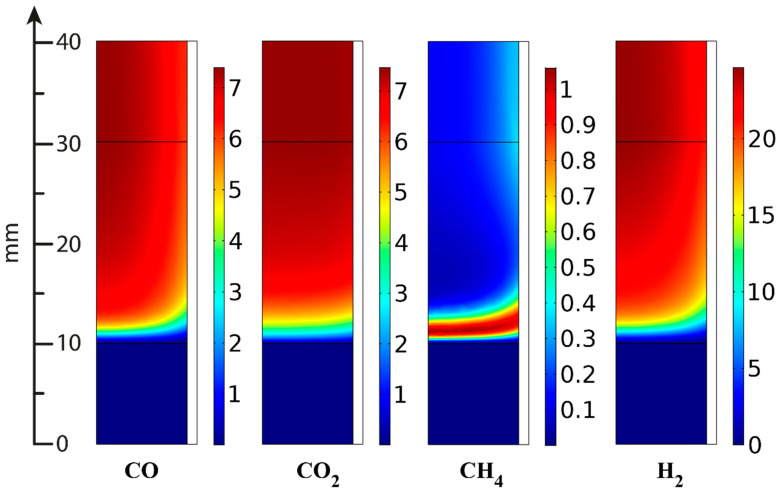
CO, CO_2_, CH_4_ and H_2_ concentrations (vol.%) distribution in ATR [95].

In the ATR experiments, the inlet gas mixture contained 8.8 vol.% oxygen, 1.35 vol.% hexadecane and 56.8 vol.% water; the rest is nitrogen. Figure 21, Figure 22 and Figure 23 present the simulation results. It is seen that oxygen (Figure 21, right) and most of the hexadecane (Figure 22, left) are consumed in a narrow region at the catalyst inlet owing to the HD oxidation reaction. This reaction is highly exothermic and maintains a high temperature in the catalyst block, peaking at around 890 °C at its frontal part. Then the temperature maximum dissipates both in the axial and radial direction both because of heat losses to the environment and the progress of the less rapid endothermic reactions. In the SR process, the central part of the block is colder than its walls because the process is supported by the reactor furnace, while the catalyst heating is limited by its radial thermal conductivity. As for the ATR process, it is supported by the heat of the reactions (mainly the HD oxidation) over the entire cross-section of the block. If we pay attention to the radial temperature gradients, they are more profound in the ATR than in the SR owing to stronger (higher) heat fluxes. Therefore, in further studies, adequate attention should be paid to an increase in the thermal conductivity of the catalytic block for ATR processes.

The C_2+_ fraction is formed in the narrow frontal part of the catalytic block (Figure 22) because of the rapid hydrogenolysis of hexadecane; then its concentration passes through a maximum and decreases owing to SR Reactions (13) and (14), facilitated by high temperatures. Here, the total yield of C_2+_ compounds is significantly lower than in the SR experiments.

Similarly, methane is formed in the narrow frontal part of the block (Figure 23) and then consumed during the reactions; its outlet concentration in ATR is lower than in the SR process. This observation correlates also with the higher temperature, which, according to reaction thermodynamics, shifts the equilibrium toward methane SR and facilitates an increase in CO and hydrogen concentrations.

Table 4 presents the calculated and experimental results of the outlet product distribution. The experimental results were averaged for the three experiments since they showed similar values of the outlet product concentrations.

Clearly, the experimental and calculated results agree well, and the equilibrium state can be achieved with a catalytic block length exceeding 300 mm.

Thus, experimental studies of diesel fuel reforming on the structured Rh/Ce_0.75_Zr_0.25_O_2_-Al_2_O_3_/FeCrAl catalytic block in the ATR and SR processes allowed the development of kinetic and mathematical models that provide a qualitatively adequate and quantitatively accurate description of experimental data [94,95,96,98].

## 8. Conclusions

In this review, a method was described for the synthesis of Rh/Ce_0.75_Zr_0.25_O_2_/Al_2_O_3_/FeCrAl catalyst for the conversion of diesel fuel into synthesis gas. Each structural component of this catalyst has its specific function. The structured metal substrate made of FeCrAl alloy provides fast heat removal/supply for exo-/endothermic reactions, possesses sufficient hydrodynamic characteristics, facilitates the manufacturing of the blocks of various geometric shapes and allows easy process upscaling. The structural oxide component (alumina) provides thermal stability and high specific surface area, strengthens mechanically the supported catalytic coating and protects the metal substrate. The active oxide component (mixed cerium–zirconium oxide with a fluorite structure) participates in the activation of water and oxygen molecules, improves the catalyst’s coke resistance through high oxygen mobility and keeps the active component in a fine dispersed state owing to strong metal–carrier interaction. The Rh nanoparticles of 1–2 nm size are involved in the activation of hydrocarbon molecules.

The synthesized catalysts were active and stable in the ATR of diesel fuel of various types, including those with a high content of aromatic hydrocarbons. The catalysts demonstrated high efficiency in the conversion of other liquid hydrocarbon fuels (gasoline and biodiesel) into synthesis gas [18,92]. The oxidative regeneration of catalysts was proved feasible; the conditions for stable catalyst operation were determined. Both the catalyst and the process are easily scalable. 

A mathematical model of the process is proposed. It provides a qualitatively adequate and quantitatively accurate description of the experimental results.

It is reasonable to focus subsequent studies on further improving the catalyst’s coke resistance and optimizing the system for high-boiling diesel fuel evaporating and mixing the steam–fuel blend with air.

## Figures and Tables

**Table 1:** A typical average composition of commercial diesel fuel.

Component	Content (vol.%)
n-Paraffins	20
iso-paraffins	15–20
Cycloparaffins	35
Alkylbenzenes	20–23
Diaromatic hydrocarbons	5
Polycyclic aromatics	<2
Sulfur compounds	0.0005–0.0008 (5–8 ppm)

**Table 2:** Qualitative and quantitative analysis of winter diesel fuel and oily residue [91].

Composition	Winter DF	Oily Residue	Dimension
Monoaromatics	25	23	wt.%
Diaromatics	5	46	wt.%
Polyaromatics	1	9	wt.%
Sulfur	8	-	ppm
H/C	1.94	-	-

**Table 3:** The optimum kinetic equations and kinetic parameters of Reactions (4)–(6) [95].

No.	Kinetic Equation *	Pre-exponential Factor $k_{i}^{0}$	Activation Energy *E_i_*, kJ/mol
1.	$W_{1}=k_{1}c_{C_{16}H_{34}}c_{O_{2}}$	1.9 × 10^6^	47.3
2.	$W_{2}=k_{2}c_{C_{16}H_{34}}c_{H_{2}O}$	5 × 10^12^	208
3.	$W_{3}=\frac{k_{3}}{c_{H_{2}}}\left(c_{CO}c_{H_{2}O}-\frac{c_{CO_{2}}c_{H_{2}}}{\exp\left(\frac{4577.8}{T}-4.33\right)}\right)$	3 × 10^8^	125
4.	$W_{4}=k_{4}\left(c_{CO}c_{H_{2}}^{3}-\frac{c_{CH_{4}}c_{H_{2}O}}{\exp\left(\frac{26800}{T}-29.8\right)}\right)$	2 × 10^2^	10
5.	$W_{5}=k_{5}c_{CO}c_{O_{2}}$	8 × 10^8^	80
6.	$W_{6}=k_{6}c_{H_{2}}c_{O_{2}}$	2 × 10^5^	40
7.	$W_{7}=k_{7}c_{C_{16}H_{34}}c_{H_{2}}^{0.5}$	3 × 10^10^	124.7
8.	$W_{8}=k_{8}\left(c_{C_{2}H_{6}}-\frac{c_{C_{2}H_{4}}c_{H_{2}}}{\exp\left(\frac{-16850}{T}+16.5\right)}\right)$	7 × 10^4^	8.4
9.	$W_{9}=k_{9}c_{C_{2}H_{6}}c_{H_{2}}^{0.1}$	1011	149
10.	$W_{10}=k_{10}c_{C_{2}H_{6}}c_{H_{2}O}^{2}$	7 × 10^13^	208
11.	$W_{11}=k_{11}c_{C_{2}H_{4}}c_{H_{2}O}^{2}$	1.5 × 10^13^	208
12.	$W_{12}=k_{12}c_{C_{2}H_{6}}c_{O_{2}}^{0.5}$	2 × 10^6^	53
13.	$W_{13}=k_{13}c_{C_{2}H_{4}}c_{O_{2}}^{0.5}$	10^6^	47

* $k_{i}=k_{i}^{0}\exp\left(-E_{i}/(R_{g}\cdot T\right))$.

**Table 4:** The outlet product distributions in the model and experimental ATP processes [95].

	HD Conversion, %	Outlet Product Distribution (vol.%)						
		CO	CO_2_	CH_4_	H_2_	N_2_	H_2_O	C_2+_
Model	99.9	6.7	7.4	0.23	23	25.5	35.9	1.1
Experiment (averaged data)	99.2	5.3	8.5	0.25	23	25.8	36.1	1.2

## Data Availability

Not applicable.

## Abbreviations

ATR—autothermal reforming; CFD—computational fluid dynamics; GHSV—gas hourly space velocity; LPG—liquefied petroleum gas; PO—partial oxidation; SR—steam reforming; SOFC—solid oxide fuel cell; MCFC—molten carbonate fuel cell; PAFC—phosphoric acid fuel cell; PEMFC—proton-exchange membrane fuel cell; DME dimethyl ether; DMM—dimethoxy methane; HD—hexadecane; DF—diesel fuel.
